# Assessment of the effect of social media use on medical students’ academic performance: cross-sectional study from Jordan

**DOI:** 10.3389/fpubh.2025.1551905

**Published:** 2025-05-13

**Authors:** Hana Taha, Diana Abu-Surrah, Luna Abu-Awadh, Ameen Mahmoud, Tamara Al-Qadi, Layan Al Hamdan, Moumen Hijazi, Abdulla Al Ani, Vanja Berggren

**Affiliations:** ^1^Department of Family and Community Medicine, School of Medicine, The University of Jordan, Amman, Jordan; ^2^Department of Neurobiology, Care Science and Society, Karolinska Institutet, Stockholm, Sweden; ^3^Department of Pharmacology, Public Health and Clinical Skills, Faculty of Medicine, The Hashemite University, Zarqa, Jordan; ^4^Office of Scientific Affairs and Research, King Hussein Cancer Center, Amman, Jordan

**Keywords:** medical students, social media, Jordan, academic performance, online interaction and engagement

## Abstract

**Background:**

The use of social media in the modern world is necessary to stay equipped with the fast-paced changes of the 21st century. The integration of social media platforms into the life of medical students and their effect on their academic performance has not been studied enough.

**Objective:**

This study examines the duration of time spent on social media, the type of content medical students is exposed to, the online engagement in study groups, and the influence of the interaction between students and their professors via social media on students’ academic performance.

**Methods:**

A cross-sectional study that used structured self-administered online questionnaire to assess the use of social media by a random sample of 429 medical students in Jordan. The data was analyzed using descriptive and multivariate analysis by SPSS 28.

**Results:**

Over 61.3% of participants used social media for 3 h or more per day. Instagram (48.7%) followed by Facebook (19.3%) and YouTube (11.9%) were the most utilized platforms. Entertainment content was the most pursued on social media (83.0%) followed by educational purposes (72.5%), sports (29.1%), and politics-related activities (17.9%). Approximately 64.6% of the students indicated that using social media is distracting, decreases their ability to focus (65.3%) and delays their daily tasks (61.8%). GPA was significantly associated with more time spent on social media (OR: 0.476; 95%CI: 0.278–0.813; *p* = 0.022). The negative impact of social networking on academic performance was significantly associated with GPA (OR: 2.292; 95%CI: 1.244–4.224; *p* = 0.007).

**Conclusion:**

This study provided evidence about the pattern and effect of using social media on the academic performance of medical students in Jordan. It delivered context-sensitive data for decision-making to enhance the positive use of social media by medical students. It also highlighted the importance of using appropriate online educational platforms to engage the medical students in relevant learning activities.

## Background

Due to the phenomenal growth of social media and its users over the past 10 years, the online world has experienced significant transformation. Social media is defined by Kaplan and Haenlein, as internet-based applications allowing creating and exchanging user-generated content (1). From 2016 to 2022, the number of active social media users worldwide increased from 2.31 to 4.62 billion (2, 3). Social media platforms, while used across almost all age groups, are most popular among adolescents and young adults. Usage of social media within the 18 to 29 years age group increased globally from 12% in 2005 to 90% in 2015 (4). These numbers bloomed with the increased usage, accessibility, and reduced costs of smartphones (5).

Social media has become an integral part of everyone’s daily life (6). It is an essential source of information and communication among students (7). Moreover, it has had a significant influence on medical students’ learning context and performance in recent years (8). Proposed by Bandura in the 1970s, the Social Learning Theory (SLT) is an overlooked theoretical framework for the impact of social media on modern day learning (9). Social media can significantly impact the learning process by influencing the individualized cognitive concepts of attention, memory, and motivation (10). In terms of attention, which is key for the internalization of knowledge, social media is able to maintain a steady stream of engagement that is purely active. This cultivates a participatory model that facilitates the sharing of user-generated knowledge strand (11). In terms of memory, social media reinforces stimuli through a variety of different graphical and audio forms. This ultimately increases the quantity and quality of symbolization and memory creation. Finally, the very presence of an online, large-scale and diverse environment enhances interaction; self-efficacy and motivation (9–11).

Social media provides fast and effective communication through dynamic settings and collaborative environments (12, 13). On the other hand, the biggest drawback of social media is its tendency to be highly addictive for most medical students (12, 13). Social media can alter the sense of reality, which makes students forget to connect with the people around them and grow emotionally distant. This obsession of being glued to their gadget screens all day brings with it an array of health disorders and can lead to stress, depression, anxiety, and sleeplessness (12, 13).

The time medical students spend on social media platforms and its effect on their academic life is still a debate. Many students spend countless hours on social media platforms which may seem like a distraction and waste of time (14) and it may also affect their Grade Point Average (GPA) by spending less time studying compared to other students who do not use social networking sites (15). Nevertheless, it may provide vital assistance to help them develop their knowledge and solve their homework. When social media is used correctly, it could contribute to the medical students’ education and professionalism. Despite that, medical ethics issues may arise, such as clouding the line between professional, personal lives and patient relation (16).

In Jordan, the number of social media users has reached 6.45 million in 2025, with 55.7% penetration rate of the total population (17). Social media’s impact on university students in general and medical students, in particular, has not been studied broadly. A study by Al-Adwan et al., an assessed social media’s impact on students’ academic performance concluded that students generally recognize social media use as helpful and can improve their learning performance (18). Most higher education students nowadays are familiar with social media use since childhood, and most of them use it profusely throughout their days (18). The students use social media for learning because study materials are uploaded across different social media platforms, which they have free and convenient access to, all the time; this, in turn, increases the level of engagement between students, their fellows and their seniors (18). In another study by Saadeh et al. that explored the usage of social media for learning by medical and dental students in northern Jordan, results showed that 59.4% of the participants believed that social media is unreliable for medical facts and information (19). In addition, the results showed variation in social media use between males and females, whereby females used greater numbers of social media applications than males and spent more time on social media. Unsurprisingly, the results also indicated that students in more advanced years of their studies relied less on social media for obtaining medical information. Furthermore, this study showed general hesitancy in relying on social media for accessing medical information, probably due to doubts about the validity of data posted across different platforms (19).

An obvious discrepancy is seen across the few studies conducted in Jordan regarding the students’ opinions on whether social media enhances academic achievements and improves academic collaboration or leaves an overall negative impact on learning and academic performance (18–20). Additionally, previous studies did not investigate the relationship between the average time spent on social media and academic performance (GPA). There is a gap of knowledge about this topic in Jordan, thus, this study aims to determine how the time spent on social media networking affects students’ academic performance. We will also identify the type of content followed by medical students and the degree of use of social media groups for collaborative studying.

## Methods

### Study setting

In 1972, Jordan established the first medical school for undergraduate medical education at The University of Jordan. After that, five other public medical schools were established. Jordan’s medical schools’ graduates are equipped with high caliber skills that are recognized globally and they are considered an essential resource for the national healthcare system (12–14). The Unified Admission Coordination Unit has listed that Jordan has almost 22,000 medical students currently studying in the Jordanian universities representing a massive student population in Jordan and still expanding (21).

### Study design

This cross-sectional study used a voluntary anonymous self-administered online structured questionnaire, hosted on Google Forms, and posted across different social media platforms to collect data from medical students at all six public universities in Jordan (23); The Hashemite University (HU), The University of Jordan (JU), The Jordan University of Science and Technology (JUST), The University of Mu’tah (MU), Yarmouk University (YU) and Al-Balqaʼ Applied University (BAU).

### Measurement tool

A structured English questionnaire was developed by the research team based on the literature (24–28) (Supplementary material 1). The questionnaire included 24 questions that were designed based on the objectives of this study. These questions covered sociodemographic characteristics; the time spent on social media; preferences of the various social media platforms; the reason for using these platforms and the effect of social media on academic performance. The questionnaire was reviewed by two experts for nitpicking and editing (29). Following that the questionnaire was pilot tested and adjusted based on 10 interviews with 5 female and 5 male students who gave feedback about the questions clarity and wording. Cronbach Alpha for the Likert-scale questions was ≥ 0.85 which confirmed the internal consistency of the questionnaire (30).

### Sampling

The questionnaire was placed on a google form and was disseminated through Facebook groups and Microsoft teams to medical students in the six public medical schools in Jordan. The data was collected from 429 undergraduate medical students, mainly aged 18–24. The inclusion criteria were: medical student enrolled in the undergraduate medical program from the first year to the sixth during the data collection period (February 2023–May 2023). The exclusion criteria were: medical student who graduated from medical school and non-medical students.

### Data analysis

Data was extracted to and cleaned within Microsoft Excel. Items with more than 20% missing responses were excluded from the final analysis. Responses to items were demonstrated in the form of descriptive statistics, mainly as frequencies and percentages for categorical variables. Associations between demographic variables, social media usage, and attitudes toward social media were explored using non-parametric two-way chi-squared test. When feasible, odds ratios (OR) and their associated 95% confidence intervals (95%CI) were included for presented associations. Data was primarily analyzed using the Statistical Package for the Social Sciences (SPSS) version 28 (IBM Corp., Armonk, N.Y., United States). Figures were produced using Graph Prism version 8.0. A *p*-value of less than 0.05 was considered statistically significant for all conducted analyses.

### Ethical considerations

This study was approved by The Hashemite University Institutional Review Board (IRB). At the beginning of the online questionnaire, the purpose of the study was explained, and a written informed consent to participate in this study was provided by the medical student. For the participants aged under 18 years, a written informed consent to participate in this study was provided by the participant legal guardian. The participants were notified that their participation is voluntary, their responses will be anonymous, confidential, and only the research team members will be able to access the data.

## Results

### Characteristics of included participants

A total of 429 medical students responded to the online questionnaire. The majority of participants were females (69.9%), are currently in their basic sciences years (66.4%), and are academically inclined (i.e., GPA rating of very good or above; 77.2%). Moreover, the greater portion of participants were sampled from the Hashemite University (71.3%), Jordan University of Science and Technology (11.4%), and the University of Jordan (6.1%). In terms of social media usage, 61.3% of participants used social media platforms for more than 120 min per day (Table 1).

**Table 1:** Characteristics of the study participants.

Variable	Category	Total
Gender	Female	300 (69.9%)
	Male	129 (30.1%)
Age	16–17	1 (0.2%)
	18–19	144 (33.6%)
	20–21	152 (35.4%)
	22–23	109 (25.4%)
	24–25	20 (4.7%)
	>25	3 (0.7%)
Year of study	1	119 (27.7%)
	2	104 (24.2%)
	3	62 (14.5%)
	4	37 (8.6%)
	5	65 (15.2%)
	6	42 (9.8%)
University	Al-Yarmouk University	14 (3.3%)
	Al-Balqa Applied University	23 (5.4%)
	JUST	49 (11.4%)
	Mut’ah University	11 (2.6%)
	The Hashemite University	306 (71.3%)
	The University of Jordan	26 (6.1%)
GPA	Fair	9 (2.1%)
	Good	89 (20.7%)
	Very good	169 (39.4%)
	Excellent	162 (37.8%)
Used social media platforms	Instagram	374 (87.2%)
	Facebook	396 (92.3%)
	YouTube	367 (85.5%)
	Twitter	177 (41.3%)
	WhatsApp	355 (82.8%)
	TikTok	122 (28.4%)
	Snapchat	238 (55.5%)
	Discord	3 (0.7%)
	Reddit	3 (0.7%)
	Telegram	4 (0.9%)
Time spent of social media	Less than 30 min	6 (1.4%)
	30 to 60 min	24 (5.6%)
	60 to 120 min	136 (31.7%)
	More than 120 min	263 (61.3%)

### Patterns of using social media platforms

**Figure 1:**
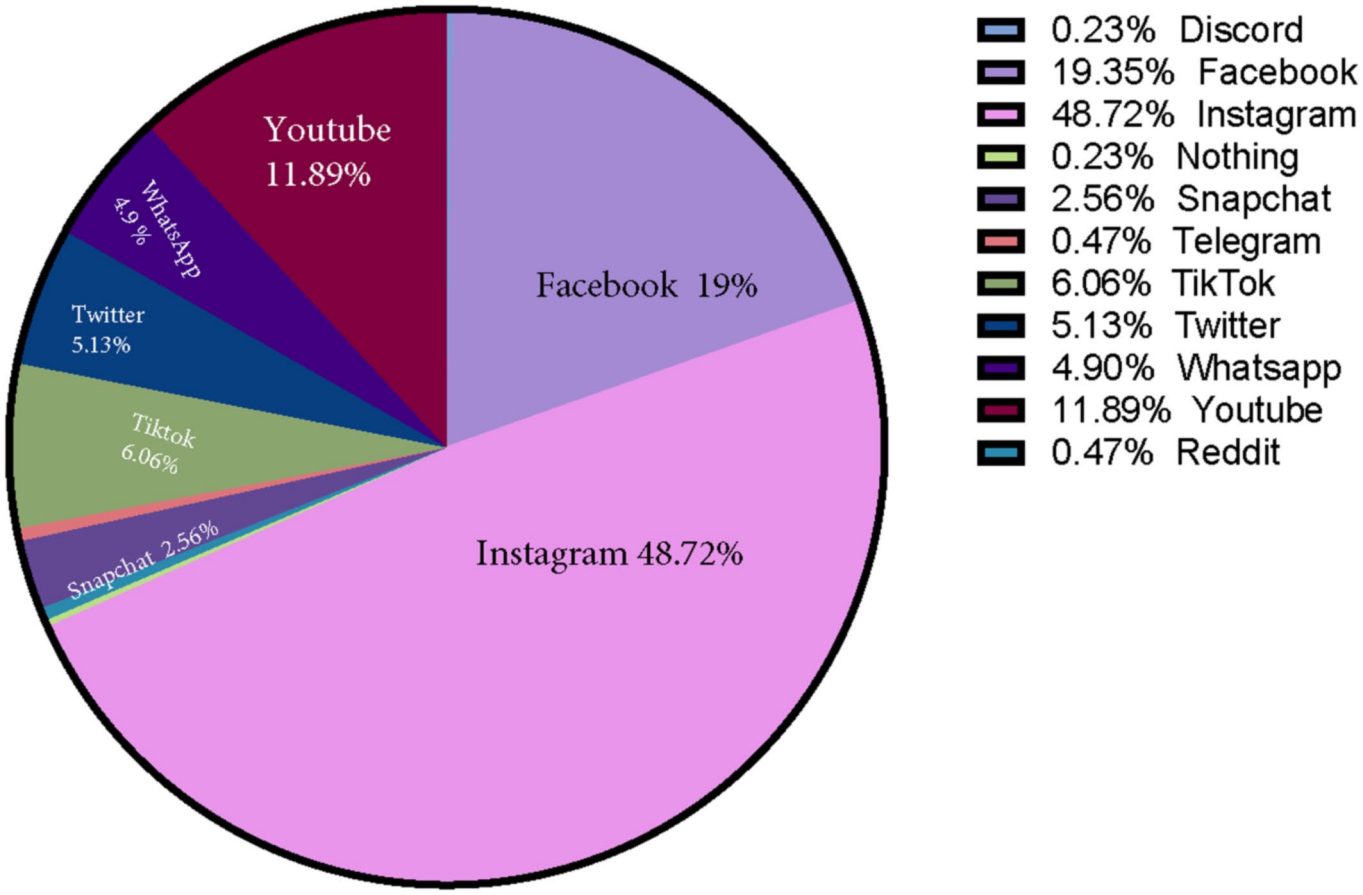
The most commonly used social media platform as rated by the students.

Instagram (48.7%) followed by Facebook (19.3%) and YouTube (11.9%) were the most followed social media platforms as rated by the students (Figure 1). In terms of followed social media content, entertainment was the most followed social media content (83.0%). Second to entertainment were educational purposes (72.5%), followed by sports (29.1%), then politics-related activities (17.9%). As for academic purposes of using social media, accessing educational courses (57.3%) and online study groups (54.8%) were the most prevalent among students. On the other hand, finding scientific journals (31.0%) or conferences (24.0%) were the least frequent. A total of 16.8% of participants declared that social media has no academic usages/benefits (Refer to Table 2). In terms of social media uses, educational and political uses did not significantly differ among genders, age groups, GPA classes, or stage of study. On the other hand, entertainment uses were significantly associated with female gender (OR: 0.522; 95%CI: 0.328–0.929; *p* = 0.024), age (OR: 2.015; 95%CI: 1.078–3.768; *p* = 0.019), and GPA (OR: 1.969; 95%CI: 1.051–3.689; *p* = 0.039). Inversely, using social media for sports-related matters was significantly associated with male gender (OR: 4.063; 95%CI: 2.600–6.348; *p* < 0.001), but not age, stage of study, or GPA.

**Table 2:** Social media usage trends among the participants.

Variable	Category	Less than 120 min	More than 120 min	*p*-value	Total
Followed social media content	Education	128 (77.1%)	183 (69.6%)	0.089	311 (72.5%)
	Entertainment	130 (78.3%)	226 (85.9%)	**0.041**	356 (83.0%)
	Political	26 (15.7%)	51 (19.4%)	0.327	77 (17.9%)
	Sports	50 (30.1%)	75 (28.5%)	0.722	125 (29.1%)
Time spent on social media affects your study time	No	29 (17.5%)	17 (6.5%)	**<0.001**	46 (10.7%)
	Yes	137 (82.5%)	246 (93.5%)		383 (89.3%)
Impact of social media	Delays tasks	88 (53.0%)	177 (67.3%)	**0.003**	265 (61.8%)
	Decreases focus	92 (55.4%)	188 (71.5%)	**0.001**	280 (65.3%)
	Increases focus	15 (9.0%)	13 (4.9%)	0.095	28 (6.5%)
	Relives stress	54 (32.5%)	87 (33.1%)	0.906	141 (32.9%)
	No effect	18 (10.8%)	11 (4.2%)	**0.007**	29 (6.8%)
Using social media affects your medical knowledge	No	33 (19.9%)	81 (30.8%)	**0.013**	114 (26.6%)
	Yes	133 (80.1%)	182 (69.2%)		315 (73.4%)
Academic social media usage purposes	Finding conferences	46 (27.7%)	57 (21.7%)	0.154	103 (24.0%)
	Educational courses	98 (59.0%)	148 (56.3%)	0.573	246 (57.3%)
	Finding scientific journals	60 (36.1%)	73 (27.8%)	0.067	133 (31.0%)
	Online study groups	88 (53.0%)	147 (55.9%)	0.559	235 (54.8%)
	No academic benefit	22 (13.3%)	50 (19.0%)	0.120	72 (16.8%)

Bold *p*-values are significant.

**Figure 2:**
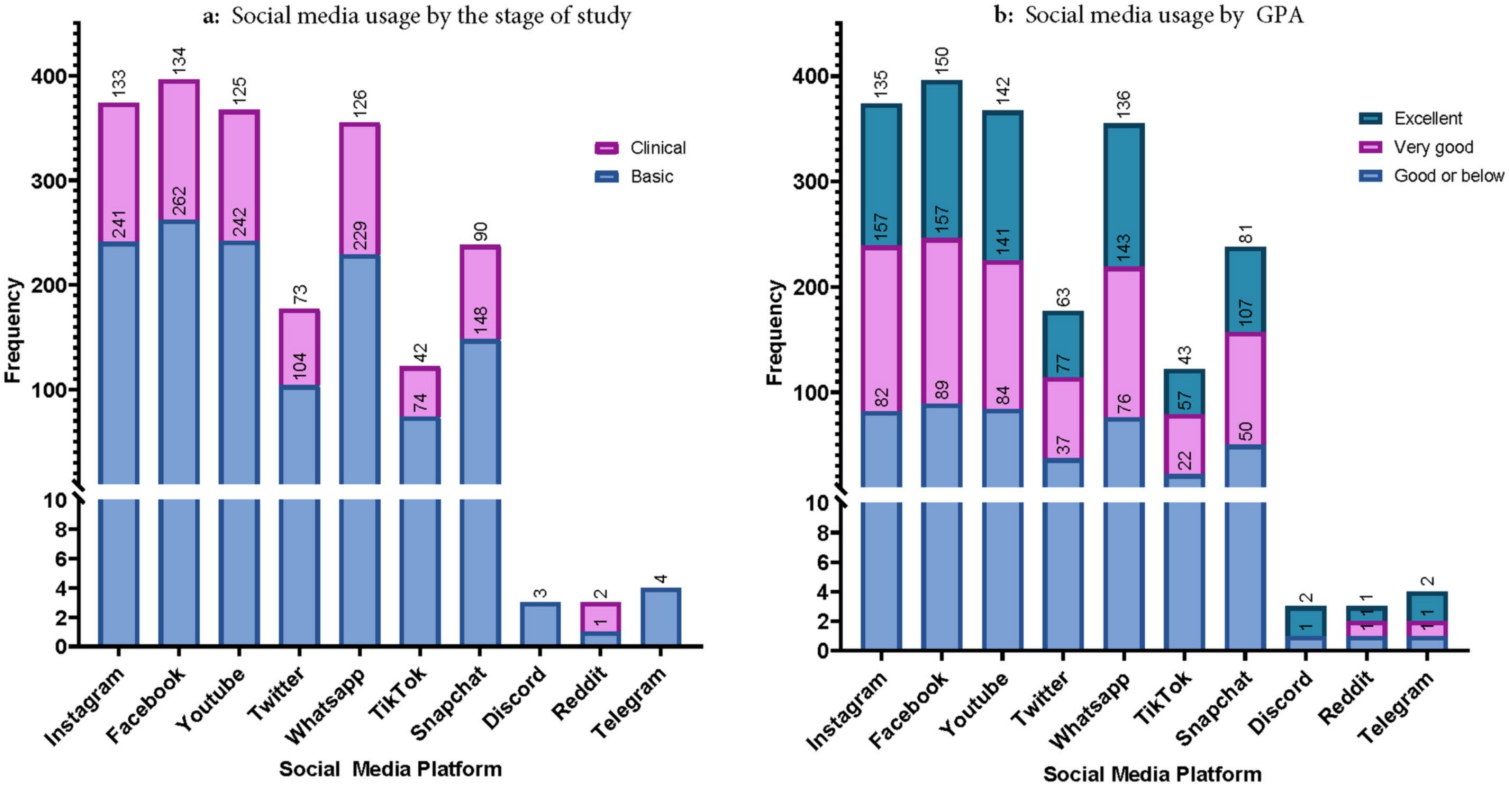
**(a)** Social media usage by the stage of study. **(b)** Social media usage by GPA.

Approximately 78% of those using Instagram, 77.5% of those using Facebook had 77% of those using YouTube had Excellent or Very Good GPA. Figures 2a,b showcases social media usage by the stage of study and GPA, respectively.

**Figure 3:**
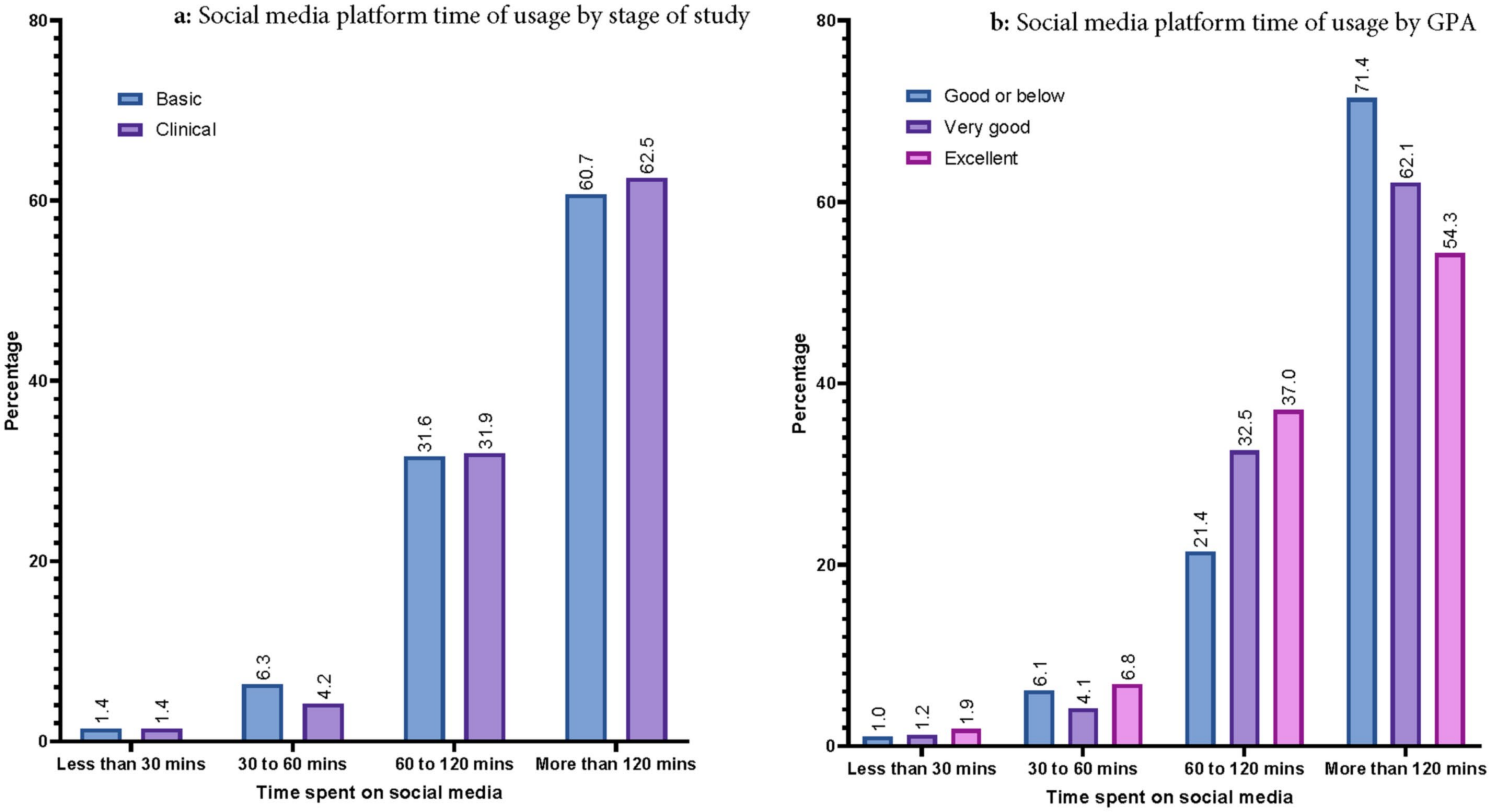
**(a)** Social media platform time of usage by stage of study. **(b)** Social media platform time of usage by GPA.

In terms of time spent on social media, females were significantly more likely to use social media more than 120 min compared to their male counterparts (OR: 0.600; 95%CI: 0.395–0.913; *p* = 0.017). Similarly, GPA was significantly negatively associated with more time spent on social media (OR: 0.476; 95%CI: 0.278–0.813; *p* = 0.022). On the other hand, age of participants and stage of study (i.e., basic vs. clinical) were not associated with time spent on social media (*p* = 0.436 and 0.718, respectively). Figures 3a,b demonstrate the time spent on social media platform by stage of study and GPA, respectively.

### Attitudes toward social media

When asked about the effects of social media on students, most of the participants agreed that social media negatively affects studying time (54.5%) and that its usage distracts them from studying (64.6%). However, the greater percentage of students concurred that social media allows for easier access of supervisors/teachers (55.0%). However, when asked about the perceived increased usage of social media during exams, a clear agreement was not reached (Refer to Table 3).

**Table 3:** Attitudes of the participates toward social media.

Variable	Category	Less than 120 min	More than 120 min	*p*-value	Total
Social media sites affect your study time negatively				0.002	
	Strongly disagree	6 (3.6%)	7 (2.7%)		13 (3.0%)
	Disagree	27 (16.3%)	22 (8.4%)		49 (11.4%)
	Neutral	59 (35.5%)	74 (28.1%)		133 (31.0%)
	Agree	55 (33.1%)	69 (26.2%)		124 (28.9%)
	Strongly agree	19 (11.4%)	91 (34.6%)		110 (25.6%)
Social media sites affect your study time positively				**<0.001**	
	Strongly disagree	27 (16.3%)	74 (28.1%)		101 (23.5%)
	Disagree	47 (28.3%)	101 (38.4%)		148 (34.5%)
	Neutral	64 (38.6%)	62 (23.6%)		126 (29.4%)
	Agree	25 (15.1%)	19 (7.2%)		44 (10.3%)
	Strongly agree	3 (1.8%)	7 (2.7%)		10 (2.3%)
Social media sites are distracting you from studying				**0.007**	
	Strongly disagree	5 (3.0%)	6 (2.3%)		11 (2.6%)
	Disagree	20 (12.0%)	19 (7.2%)		39 (9.1%)
	Neutral	49 (29.5%)	53 (20.2%)		102 (23.8%)
	Agree	58 (34.9%)	85 (32.3%)		143 (33.3%)
	Strongly agree	34 (20.5%)	100 (38.0%)		134 (31.2%)
Social media helps you reach out to your supervisor/teacher in an easier way				0.511	
	Strongly disagree	10 (6.0%)	24 (9.1%)		34 (7.9%)
	Disagree	23 (13.9%)	33 (12.5%)		56 (13.1%)
	Neutral	36 (21.7%)	67 (25.5%)		103 (24.0%)
	Agree	51 (30.7%)	75 (28.5%)		126 (29.4%)
	Strongly agree	46 (27.7%)	64 (24.3%)		110 (25.6%)
Social media use increases during exams				0.055	
	Strongly disagree	33 (19.9%)	39 (14.8%)		72 (16.8%)
	Disagree	42 (25.3%)	50 (19.0%)		92 (21.4%)
	Neutral	37 (22.3%)	65 (24.7%)		102 (23.8%)
	Agree	37 (22.3%)	53 (20.2%)		90 (21.0%)
	Strongly agree	17 (10.2%)	56 (21.3%)		73 (17.0%)
Social networking has impacted your academic performance				**<0.001**	
	Negative	65 (39.2%)	169 (64.3%)		234 (54.5%)
	No effect	55 (33.1%)	56 (21.3%)		111 (25.9%)
	Positive	46 (27.7%)	38 (14.4%)		84 (19.6%)
The integration of social media with educational platforms facilitated the education process				0.372	
	Disagree	19 (11.4%)	38 (14.4%)		57 (13.3%)
	Agree	147 (88.6%)	225 (85.6%)		372 (86.7%)
Integration of social media within the education process introduces privacy concerns				**0.045**	
	Disagree	84 (50.6%)	159 (60.5%)		243 (56.6%)
	Agree	82 (49.4%)	104 (39.5%)		186 (43.4%)
Academic staff are capable of integrating social media into educational platforms				0.377	
	Disagree	76 (45.8%)	109 (41.4%)		185 (43.1%)
	Agree	90 (54.2%)	154 (58.6%)		244 (56.9%)
Social media aids group work				0.295	
	Disagree	21 (12.7%)	43 (16.3%)		64 (14.9%)
	Agree	145 (87.3%)	220 (83.7%)		365 (85.1%)
Social medial aids in collaborative work				0.807	
	Disagree	75 (45.2%)	122 (46.4%)		197 (45.9%)
	Agree	91 (54.8%)	141 (53.6%)		232 (54.1%)
How has social media affected your research skills?				**0.018**	
	Negatively	104 (62.7%)	128 (48.7%)		232 (54.1%)
	No effect	30 (18.1%)	67 (25.5%)		97 (22.6%)
	Positively	32 (19.3%)	68 (25.9%)		100 (23.3%)

Enhancing access to supervisors, and the increased usage of social media during exams were not associated with gender, age, GPA, or stage of study. Overall, most of the students believed that social networking had a negative impact on their academic performance (54.5%) and research skills (54.1%). On another note, the majority of students agreed that the integration of social media is able to facilitate the education process (86.7%) and that academic staff can utilize such integration to enhance learning (56.9%). Students were also appreciative of the role of social media in facilitating group work (85.1%) and collaborative work (54.1%).

The negative impact of social media on study time was perceived significantly greater by females compared to males (*p* = 0.036). There were no significant differences in such perception among different age, GPA, or stage groups. On the other hand, the impact of social networking on academic performance was significantly associated with different GPA groups (OR: 2.292; 95%CI: 1.244–4.224; *p* = 0.007) but not age, gender, or stage of study. However, the impact of social media on knowledge of medicine was not associated with age, GPA, gender, or stage of study.

Females were significantly more likely to use social media for online studying groups (OR: 1.760; 95%CI: 1.161–2.673; *p* = 0.007) while males declared that social media provides no academic/scholarly benefits (OR: 1.861; 95%CI: 1.104–3.139; *p* = 0.019). Higher GPA was associated with the perception that social media usage can facilitate the educational process (OR: 3.515; 95% CI: 1.686–7.327; *p* = 0.002). In contrast, the introduction of privacy concerns by social media was not associated with age, gender, GPA, or stage of study. In terms of the perceived readiness of academic staff to integrate social media within the educational system, females and students in their basic years were more likely to indicate that academic staff are not optimal users of social media (OR: 1.887; 95%CI: 1.224–2.907; *p* = 0.004 and OR: 1.692; 95%CI: 1.118–2.561; *p* = 0.013, respectively). Similarly, females were significantly more likely to perceive that social media enables group work (OR: 2.032; 95%CI: 1.179–3.508; *p* = 0.010) compared to their male counterparts. Finally, younger students and those in their basic years were significantly more likely to declare that social media facilitated collaborative work (OR: 2.132; 95%CI: 1.418–3.205; *p* < 0.001).

## Discussion

The results of this study showed that time spent daily on social media was 3 h or more by most of the participants. Instagram followed by Facebook were the most followed social media platforms as rated by the students. Entertainment content was the most pursued on social media (83.0%) followed by educational purposes (72.5%), sports (29.1%), and politics-related activities (17.9%). These findings are consistent with regional literature. Al Faris et al., in their exploration of the impact of social media on medical students in Saudi Arabia demonstrated that the majority of students (55%) spent 1–4 h a day on social media, while 23% spent more than 4 h (31). The main reasons for social media use were entertainment (95.8%), staying up to date with news (88.3%), socializing (85.5%) and for academia-related purposes (40%). The most popular applications used for learning purposes were YouTube (83.5%), WhatsApp (35.5%) and Twitter (35.3%). Another Saudi study conducted in Jazan university (24) found that the most common internet website e used by medical students was Facebook (53%). The participants used social media for 2 to 4 h daily and 65.9% of them used social networks for more than 3 years. In a study conducted in Jordan by Al-Adwan et al., 2020, learning was one of the main reasons for using social media by the students, mainly because study materials are uploaded at different social media platforms (18). According to another study in Nigeria (32), social media was used by the students for entertainment, networking, and academic purposes. In a study conducted in India by Sobaih et al., half of the male students used social media for personal reasons, such as keeping in touch with friends and family. However, a large percentage of female respondents used it for entertainment purposes (33).

In our study 64.6% of the students indicated that using social media is distracting, decreases their ability to focus (65.3%) and delays their daily tasks (61.8%). This could be attributed to using social media for entertainment purposes rather than educational ones. Using social media excessively can lead to a lack of focus on learning and decreased academic performance; it cultivates unhealthy habits that influence studying and academic performance, namely, difficulty completing tasks, troubled sleep patterns and dietary problems. So, non-heavy users have more satisfactory grades than heavy users (34, 35).

In a study conducted in Ghana found that most of the students had access to the internet on their mobile phones and were aware of the presence of social media sites. As a result, they visit their social media sites and spend between 30 min to 3 hours every day. This had negative effects on the academic performance of the respondents. Furthermore, the study stated that the students’ social networking has grown in popularity over time and was used to contact individuals outside of school. Although people might feel a sense of community through social networking (36), still, inappropriate social media behavior can be detrimental to students’ future opportunities (37).

In our study, participants with higher GPA were less likely to spend more than 120 min on social media compared to their counterparts with lower GPA ranks. Moreover, those with higher GPA were more likely to declare that social media has a negative impact on their academic performance. These results are consistent with the body of regional literature as demonstrated in Saudi Arabia, Iraq, and Lebanon (28, 38, 39). Such a phenomenon could be simply attributed to the strong correlation between academic performance and self-esteem. Students with higher GPA typically have high self-esteem and are more likely to be more motivated to complete academic tasks irrespective of external distractors. The relationship between lower academic performance and social media addiction has been observed in the literature (40). The excessive use of social media contributes to an imbalance in the time economy of students. Across both undergraduate, secondary, and primary levels, higher usage of social media or internet was associated with less time studying, ultimately worsening academic performance (40).

It appears that students with pre-existing academic problems may develop unhealthy social media usage tendencies as a mean to mitigate their social anxiety and satisfy their need for social reassurance (41). Procrastination due to the distracting nature of social is also implicated to negatively affect students’ attitudes toward schoolwork. Interestingly, we could expand the SLT model to account for social media impact on improving learning by increasing the opportunities for priming, memory formation, and motivation (9, 10). However, in practice, the near-infinite sources of information may overwhelm students. Similarly, their social identities and sense of self may get blurred in the noisy cyber space while networking and interacting with many other students (42).

Yet, social media has consistently shown a positive impact on learning and performance through enhancing students’ relationships, improving their motivation, developing their collaborative abilities, and offering a personalized learning platform (43). A study in Babylon university in Iraq reported that 42% of the student’s academic performance was affected positively by social media (28). The positive effects were attributed to browsing medical pages, studying in groups, and interacting with classmates; however, the negative effects were attributed to excessive time spent on Facebook and other social media for non-academic purposes, distractions, and a lack of control. A study by Mohammed Habes et al., 2018, from Yarmouk University in Jordan focused on the use of YouTube and its effects on higher education students’ academic performance (44). It recognized YouTube as a powerful and helpful means of learning and pointed out that most young people and university students have YouTube accounts these days. However, it stressed that high level of addiction to social media might distract students from executing their academic tasks in the usual manner, as they can contact each other instantly for different reasons and share information heavily (6, 44).

Many researchers found a positive association between social media networking and students’ academic performance (28, 45). A study in Iraq conducted by the college of medicine at Babylon university (28), reported that 42% of the student’s academic performance was affected positively by social media. On the other hand, 33% were affected negatively, and the rest, 25%, reported no effect of social media on their academic performance. The two main reasons for using social networking websites were visiting medical pages and groups (57.9%), communicating with friends and following their posts and updates (54.4%). Other reasons were killing spare time (45.6%), studying in groups on Facebook messenger (40.4%), news (38.6%), fashion and styles (22.8%) and sports (15.8%). Those who reported positive effects of social media attributed this to the ability to browse medical pages, study with groups, and interact socially with other students. On the contrary, those who reported negative effects complained of excessive time spent on Facebook and other social media for non-academic purposes, while those who stated no influence of social media on grades reported controlled use for social media in balance with their study time.

This study provides valuable insights into social media usage and academic performance of medical students; however, there are several limitations that should be considered. A predominant proportion of our participants were from the Hashemite University of Jordan, primarily in their first year. Potentially limiting the generalizability of our results to a broader population of medical students in Jordan. Additionally, the participants’ responses to questions about the effects of social media on their academic performance were self-reported. As such, they may be subject to recall bias or social desirability bias. Our study is cross sectional; it provided insights into associations between social media use and academic performance but did not establish causality. We examined social media use and academic performance at a specific time point, so long-term trends or fluctuations in students’ social media behavior may not be accurately captured. Finally, not all factors influencing the relationship between social media and academic performance were accounted for. Such factors may include but are not limited to previous academic performance, cognitive capacity, emotion regulation, and psychological burden (e.g., depression).

## Conclusion

Medical students use social media tools in various innovative ways for entertainment, education and peer support, but social media still holds a potential risk, therefore, medical students should be aware of social media’s benefits and drawbacks to make the best decisions for themselves. While this study provides valuable insights into the impact of social media on the academic performance of medical students, there is still much to learn about the intricacies of this relationship. Future research endeavors can build upon these findings to develop a more comprehensive understanding of the dynamics between social media use and academic performance among students in the healthcare field.

### Recommendations and future direction

Using social media platforms by medical students is beneficial in communication and education. Nevertheless, it is essential to recognize the drawbacks of social networking, therefore, educational institutions should make it easier for students to obtain trustworthy and organized information which can help them in their academic work and make communication between the students and professors easier (46). The association between social media and academic performance should be tackled through longitudinal research which uses objective measures of both academic performance and daily social media usage trends. Investigating the role of time management skills, content consumption patterns, and individual differences may shed more light on this complex relationship. Additionally, there is a need for qualitative studies to explore in depth the medical students’ perceptions and attitudes toward social media.

The use of social media continues to evolve, future studies may need to adapt their methodologies to account for changes in platform popularity, features, and usage patterns. Exploring the potential effectiveness of tailored interventions that aim to promote responsible social media use by medical students, could also be a valuable avenue for future research.

## Data Availability

The raw data supporting the conclusions of this article will be made available by the authors, without undue reservation.
