# Supplementary material for: Assessment of the effect of social media use on medical students’ academic performance: cross-sectional study from Jordan
Source: Front Public Health. 2025 May 13;13:1551905. doi: 10.3389/fpubh.2025.1551905 (PMC12106426; doi:10.3389/fpubh.2025.1551905)
Supplement: Supplementary file 1 [file Data_Sheet_1.PDF]

Supplement 1: Measurement tool

Assessment of Social Media's Impact on Medical Students' Academic Performance: Cross-sectional Study from Jordan

This study aims to examine the duration of time spent on social media by medical students from Jordanian universities, the type of content followed by them and its impact on their academic performance. The data is anonymous and will be used for research purpose only. Your participation is voluntary, and you have the full autonomy to withdraw and stop your participation at any time.

I consent to participating in this research knowing that all the information that I will provide will be confidential and for research purposes only. **For those under the age of 18, one of your legal guardians must consent before you participate in this research**

1. Age

16-17

18-19

20-21

22-23

24-25

>25

I agree to participate ☐ **legal guardian agree that you participate** ☐

2. Gender

Male

Female

3. Year of Study

1

2

3

4

5

6

4. University:

1. The Hashemite University
2. The University of Jordan
3. Jordan University of Science & Technology
4. Al Yarmouk University
5. Mu'tah University
6. Al-Balqa Applied University

5. GPA

1. Excellent
2. Very Good
3. Good
4. Fair

6. Do you use social media platforms?

a. Yes
b. No

7. What social media sites do you use?

a. Instagram
b. Facebook
c. YouTube
d. Snapchat
e. Twitter
f. WhatsApp
g. TikTok
h. Other:

8. Which social media site do you use the most?

a. Instagram
b. Facebook
c. YouTube
d. Snapchat
e. Twitter
f. WhatsApp
g. TikTok
h. Other:

9. The main reason behind your social media use?

a. Entertainment
b. Education
c. News
d. Communication and making friends
e. Blogging
f. Other

10. How much time do you spend on social media daily?

a. Less than 30 minutes
b. 30 minutes to an hour
c. One to two hours
d. 3 hours or more

11. What type of content do you follow?

a. Educational
b. Entertainment
c. Political
d. Sports
e. Other

12. Does the time you spend on social media effect you?

a. Yes
b. No

13. How does it affect you?

a. It decreases my ability to focus while studying
b. It increases my ability to focus while studying
c. Delays my daily tasks and assignments
d. Relieves academic stress
e. No effect

14. Do you think social media improves your knowledge of medicine?

a. Yes
b. No

15. Do you think social media sites affect your study time negatively?

Strongly Disagree Strongly Agree

1 2 3 4 5

16. Do you think social media sites affect your study time positively?

Strongly Disagree Strongly Agree

1 2 3 4 5

17. Do you feel social media sites are distracting you from studying?

Strongly Disagree Strongly Agree

1 2 3 4 5

18. Do you think social media helps you reach out to your supervisor/teacher in an easier way?

Strongly Disagree Strongly Agree

1 2 3 4 5

19. Do you think your social media use increases during exams?

Strongly Disagree Strongly Agree

1 2 3 4 5

20. Do you think social networking has impacted your academic performance?

a. A positive impact
b. A negative impact
c. It had no impact on my performance

21. In which of the following did social media help you engage in?

a. Online study groups
b. Scientific journals
c. Conferences
d. Educational courses
e. I haven't benefited from social media

22. In your opinion, has the integration of social media with educational platforms facilitated the education process?

a. Yes, the learning process has been easier
b. No, it did not facilitate the education process

23. Do you have concerns regarding your privacy due to the integration of social media and educational platforms?

a. Yes, I do not favor this integration due to privacy concerns
b. I don't have any privacy concerns

24. Where do you think the academic staff stand currently regarding the integration of social media and educational platforms?

a. The academic staff don't know how to use these platforms very well yet
b. The academic staff are well informed and trained in using these platforms

25. Do you think social media sites aid group work?

a. Yes
b. No

26. What was your own experience about the use of social media for collaborative work?

a. Collaborative learning experience in the social media environment is better than in a face-to-face
b. Social media has created boundaries regarding group work

27. How has social media affected your research skills?

a. I have been able to develop my research skills through collaboration with my peers and colleagues
b. I haven't benefited from social media regarding my research skills
c. I have no interest in the research field
